# Exercise and diabetes have opposite effects on the assembly and O-GlcNAc modification of the mSin3A/HDAC1/2 complex in the heart

**DOI:** 10.1186/1475-2840-12-101

**Published:** 2013-07-09

**Authors:** Emily J Cox, Susan A Marsh

**Affiliations:** 1 Graduate Program in Nutrition and Exercise Physiology, College of Pharmacy, Washington State University, Spokane, WA, USA; 2 Program in Nutrition and Exercise Physiology, College of Pharmacy, Washington State University, PO Box 1495, Spokane, WA 99210-1495, USA

**Keywords:** Exercise, Diabetes, Cardiac hypertrophy, O-GlcNAc, Fetal genes

## Abstract

**Background:**

Exercise causes physiological cardiac hypertrophy and benefits the diabetic heart. Mammalian switch-independent 3A (mSin3A) and histone deacetylases (HDACs) 1 and 2 regulate hypertrophic genes through associations with the DNA binding proteins repressor element-1 silencing transcription factor (REST) and O-linked β-N-acetylglucosamine transferase (OGT). O-linked β-N-acetylglucosamine (O-GlcNAc) is a glucose derivative that is chronically elevated in diabetic hearts, and a previous study showed that exercise reduces cardiac O-GlcNAc. We hypothesized that O-GlcNAc and OGT would physically associate with mSin3A/HDAC1/2 in the heart, and that this interaction would be altered by diabetes and exercise.

**Methods:**

8-week-old type 2 diabetic *db/db* (*db*) and non-diabetic C57 mice were randomized to treadmill exercise or sedentary groups for 1 or 4 weeks.

**Results:**

O-GlcNAc was significantly higher in *db* hearts and increased with exercise. *Db* hearts showed lower levels of mSin3A, HDAC1, and HDAC2 protein, but higher levels of HDAC2 mRNA and HDAC1/2 deacetylase activity. Elevated HDAC activity was associated with significantly blunted expression of α-actin and brain natriuretic peptide in *db* hearts. In sedentary *db* hearts, co-immunoprecipitation assays showed that mSin3A and OGT were less associated with HDAC1 and HDAC2, respectively, compared to sedentary C57 controls; however, exercise removed these differences.

**Conclusions:**

These data indicate that diabetes and exercise oppositely affect interactions between pro-hypertrophic transcription factors, and suggest that an increase in total cardiac O-GlcNAc is a mechanism by which exercise benefits type 2 diabetic hearts.

## Background

Cardiac hypertrophy is the adaptive enlargement of the myocardium in response to physical or neurohormonal stress. Type 2 diabetes is associated with a cardiac syndrome called diabetic cardiomyopathy, which is characterized by pathological hypertrophy, contractile dysfunction [1,2], and an intractable reliance on fatty acid oxidation [3-5]. By contrast, chronic endurance exercise training causes physiological hypertrophy that improves contractile mechanics [6,7] and myocardial metabolism [8,9]. Exercise benefits the type 2 diabetic heart [10-14], but the underlying mechanisms by which exercise and diabetes control cardiac hypertrophy are not well understood.

In non-diabetic hearts, activation of fetal genes is a protective mechanism [15-17] that accompanies pathological hypertrophy [17-21]. These fetal genes include fetal cytoskeletal proteins (skeletal α-actin, β-myosin heavy chain) and the atrial and brain natriuretic peptides (ANP and BNP) [22-24]. Importantly, exercise and diabetes moderate these genes differently. Exercise increases adult cardiac α-actin [25], but does not change fetal gene expression [26], whereas type 2 diabetes actually reduces circulating natriuretic peptides [27,28], and blocks the activation of fetal genes by hypertrophic stimuli in vitro [29]. This suggests that fetal gene regulation in diabetic hearts is different from that of exercised hearts and non-diabetic hearts, and therefore, may underlie the hypertrophic response to these conditions.

A potential mechanism for the effects of diabetes and exercise on fetal genes is through the post-translational modification of transcription factors by O-linked β-N-acetylglucosamine (O-GlcNAc). O-GlcNAc is a glucose derivative that post-translationally modifies serine/threonine residues [30,31]. O-GlcNAcylation modifies signal transduction in a manner analogous to phosphorylation; O-GlcNAc transferase (OGT) and O-GlcNAcase (OGA) add and remove the O-GlcNAc moiety, respectively. We have recently shown that O-GlcNAc modifies repressor element-1 silencing transcription factor (REST) [32], a transcription factor that represses fetal genes by recruiting the corepressors mammalian switch-independent 3A (mSin3A) and histone deacetylases (HDACs) 1 and 2 [33]. The HDAC enzymes deacetylate histone tails, thus condensing euchromatin and silencing gene expression; HDAC1 and HDAC2 specifically mediate fetal gene regulation by REST and mSin3A, and have been repeatedly linked to hypertrophic growth of the heart [34].

mSin3A and HDAC1 are O-GlcNAcylated in HepG2 liver carcinoma cells, and are recruited to gene loci by the OGT enzyme [35], which also O-GlcNAcylates itself [30]. The activity of OGT is regulated by cellular concentrations of UDP-GlcNAc substrate [36,37], which is increased in the diabetic heart [38]. Indeed, total O-GlcNAc and protein O-GlcNAcylation are elevated in the diabetic heart [4,39], but the functional implications of this finding are unclear as elevated cardiac O-GlcNAc is implicated in heart failure and cardiac dysfunction [39,40], but also cardioprotection [41,42]. Nevertheless, two previous studies showed that total protein O-GlcNAcylation and O-GlcNAcylation of the SP1 transcription factor are lowered by swimming exercise in both non-diabetic and streptozotocin-induced type 1 diabetic hearts [43,44], and OGA overexpression directly ameliorates the cardiovascular complications of type 2 diabetes [38].

We therefore hypothesized that a reduction in O-GlcNAc may be a mechanism by which exercise benefits the type 2 diabetic mouse heart. However, since these interactions have not been studied in the heart, the secondary purpose of this study was to characterize the effects of diabetes and moderate exercise on the mSin3A/HDAC1/2 complex. We used 4 weeks of moderate treadmill exercise training to investigate the early signaling mechanisms in the hypertrophic process. We show that exercise increases total protein O-GlcNAcylation in the type 2 diabetic *db*^*+*^*/db*^*+*^ mouse heart, and that exercise and diabetes have reciprocal effects on the association of HDAC1 and HDAC2 with fetal gene-regulating transcription factors.

## Methods

### Animal care and facilities

The procedures in this study followed the guidelines of the Washington State University Institutional Animal Care and Use Committee and the Guide for the Care and Use of Laboratory Animals published by the National Institutes of Health (NIH publication no. 85–23, revised 1996). 8-week-old type 2 diabetic mice (B6.BKS(D)-*Lepr*^*db*^/J, *db*^*+*^*/db*^*+*^ (*db*) and age-matched C57BL/6J non-diabetic lean *db*^*+*^*/?* background strain controls (C57) were purchased from Jackson Laboratories (Bar Harbor, ME). To control for activity, mice were singly housed without environmental enrichment in a climate-controlled vivarium on a 12:12 light:dark cycle. Mice consumed water and standard chow ad libitum, except for one overnight fast per week prior to blood glucose measurement.

### Exercise training protocol

Mice ran on a 6-lane electric treadmill (Columbus Instruments, Columbus, OH) for 5 consecutive days a week with 2 days of rest. In week 0, all mice were acclimated to the treadmill by standing on the stationary belt for 10 min, then walking at 5 m/min for 20 min. Mice were then randomized to sedentary (n = 11) or exercise (n = 12) groups for 1 week, or sedentary (n = 16) or exercise (n = 15) groups for 4 weeks. Human patients with type 2 diabetes are commonly prescribed an exercise intensity of at least 40-60% of their aerobic capacity, but a higher intensity is recommended for maximum health benefits [45]. Therefore, we exercised mice at 10 m/min, which corresponds to approximately 70% of maximal oxygen uptake (VO_2_max) for the C57 strain [46]. Mice ran at this speed at 0% grade for 10, 20, 30, or 40 min in weeks 1, 2, 3, and 4, respectively. O-GlcNAc is a highly dynamic stress response; therefore, to remove confounding effects of stress, we kept the treadmill covered, and used gentle tactile stimuli rather than the electroshock apparatus to keep mice running. Sedentary groups were handled identically and spent equal time in the same treadmill environment on a stationary belt.

### Blood glucose and body weight measurements

Blood glucose and body weight were measured weekly after an overnight fast. Blood glucose was measured with a glucometer (ACCU-CHEK® Aviva, Roche Diagnostics, Indianopolis, IN) in a small sample of tail blood. Glucose readings that exceeded the accuracy limit of the calibrated meter (33.3 mmol/L) were imputed this value for statistical analysis.

### Tissue harvesting and morphological measurements

Following an overnight fast, mice were anesthetized rapidly using an isoflurane vaporizer chamber with 2-4% isoflurane gas in 100% oxygen. Mice were immediately decapitated and blood glucose was measured in neck blood. Whole hearts were excised and the atria were removed. Ventricular tissue was immediately wet-weighed, cut into four equal tissue aliquots, snap-frozen in liquid nitrogen, and stored at −80°C. The left tibia was dissected from each animal and measured from the tibial plateau to the lateral malleolus.

### Western blotting

Ventricular tissue was homogenized in Tissue Protein Extraction Reagent (Sigma-Aldrich, St. Louis, MO); 20 mM sodium fluoride; 1 mM sodium orthovanadate; 3% protease inhibitor cocktail (Sigma); and 0.02% PUGNAc, an OGA inhibitor (Sigma), to inhibit O-GlcNAc removal from proteins. Total protein was quantified with a modified Lowry assay (BioRad, Hercules, CA). Proteins were separated by SDS-PAGE and transferred onto PVDF membranes, which were probed overnight at 4°C with primary antibodies (anti-HDAC1 and -HDAC2, Cell Signaling, Beverly, MA; anti-mSin3A, -REST, -OGT, and -calsequestrin, Abcam, Cambridge, MA; anti-NCOAT/OGA, Santa Cruz Biotechnology, Santa Cruz, CA; anti-phospho-HDAC1 (Ser 421/423), Millipore, Billerica, MA), then probed with the appropriate secondary antibodies for 1 hour at room temperature (see Table 1 for antibody details). Chemiluminescent substrates (Thermo Fisher Scientific, Rockford, IL) were used to detect horseradish peroxidase activity on a ChemiDoc (BioRad). Protein levels were quantified on duplicate blots with standard densitometry using ImageJ software (National Institutes of Health, Bethesda, MD), and normalized to the loading control calsequestrin.

**Table 1:** Antibodies

**Target**	**Supplier**	**Cat #**
CTD 110.6 (anti-O-GlcNAc)	Gift from Mary-Ann Accavitti, University of Alabama at Birmingham	n/a
RL2 (anti-O-GlcNAc)	Abcam, Cambridge, MA	2739
anti-mSin3A	Abcam	3479
anti-OGT	Abcam	50271
anti-NCOAT/OGA	Santa Cruz Biotechnology, Santa Cruz, CA	sc-66612
anti-HDAC1	Cell Signaling, Beverly, MA	5356
anti-HDAC2	Cell Signaling	5113
anti-Calsequestrin	Abcam	3516
anti-REST	Abcam	21635
anti-phospho-HDAC1 (ser421/423)	Millipore, Billerica, MA	07-1575

### O-GlcNAc Western blotting

Ventricular lysates were separated with SDS-PAGE and transferred to nitrocellulose membranes. Membranes were blocked overnight at 4°C, probed with 1:5000 anti-O-GlcNAc antibody (CTD 110.6, generous gift of Mary-Ann Accavitti, University of Alabama at Birmingham), then probed with the appropriate secondary antibody. Horseradish peroxidase activity was detected on x-ray film with chemiluminescent substrate (Thermo Scientific) and quantified as described above for Western blotting.

### Co-immunoprecipitation

Ventricular tissue was homogenized in Tissue Protein Extraction Reagent, 1% phosphatase inhibitor, 2% protease inhibitor (Sigma), and 0.02% PUGNAc. Lysates were assayed for total protein as described for Western blotting. Samples were diluted to equal protein concentrations and precleared over protein A/G agarose beads (Thermo Fisher Scientific) at 4°C for 4 hours. Precleared supernatants were then added to 25 ul of beads that had been incubated with primary antibody for 4 hours at 4°C. IP was performed overnight at 4°C; beads were then washed and eluted at 100°C for 5 min. The eluents were assayed for co-immunoprecipitated proteins using immunoblotting. The positive control was ventricular lysate; the negative control was ventricular lysate that was immunoprecipitated without antibody. Co-immunoprecipitated proteins were normalized to the level of captured target protein for analysis.

### HDAC activity

HDAC enzyme activity was assayed with a colorimetric kit (Enzo Life Sciences, Farmingdale, NY) per the manufacturer’s instructions. The substrate for this assay is predominantly deacetylated by HDAC1/2 and sirtuin 1 (a class III HDAC), but not the class II HDACs. Optical density was read at 415 nm. Results are presented as fold changes from control absorbance.

### RNA isolation and quantitative real-time PCR (qPCR)

RNA was isolated with an RNA isolation kit (Qiagen, Valencia, CA) and quantified on a NanoPhotometer™ spectrophotometer (Implen, Ontario, NY). Complementary DNA (cDNA) was generated using a cDNA synthesis kit (Thermo Fisher Scientific). qPCR was performed in triplicate with SYBR green fluorescence chemistry using a qPCR kit (Qiagen). The negative control contained water in place of cDNA template. Thermal cycling was performed on an iCycler iQTM Real Time PCR Detection System (Biorad) using the following cycle: 95°C for 10 min, and 40 cycles of 95°C for 30 sec and T_m_ for 10 sec. Primer specificity was confirmed by melting curve analysis. Amplification data were analyzed with the 2^-ΔΔ*Ct*^ method for normalization to the housekeeping gene GAPDH as previously described [47]. See Table 2 for primer details.

**Table 2:** Real-time PCR primers

**Primer**	**Forward**	**Reverse**	**Amplicon**	**Annealing**
			**size (kb)**	**temp (°C)**
ANP	TTCCGGTACCGAAGATAACAGCCA	TGACACACCACAAGGGCTTAGGAT	91	60
BNP	AGACAAGGGAGAACACGGCATCAT	ACAGAATCATCTGGGACAGCACCT	85	60
HDAC1	TTCCTGCGTTCTATTCGCCCAGAT	AACAAGCCATCAAACACCGGACAG	98	60
HDAC2	TACAACAGATCGCGTGATGACCGT	TCCCTTTCCAGCACCAATATCCCT	94	62
α-skeletal actin	TTGTGCACCGCAAATGCTTCTAGG	GCAACCACAGCACGATTGTCGATT	90	60
α-cardiac actin	TGTAGGTGATGAAGCCCAGAGCAA	TGGTGCCAGATCTTCTCCATGTCA	105	60
β-myosin heavy chain	TGGCTGGTGAGGTCATTGACAGAA	TGGCTGGTGAGGTCATTGACAGAA	104	60
GAPDH	TGTGATGGGTGTGAACCACGAGAA	CATGAGCCCTTCCACAATGCCAAA	133	Per plate

### Statistics

Longitudinal effects of genotype and exercise on body weight and blood glucose were analyzed with two-factor repeated measures ANOVA followed by Bonferroni post-hoc tests. Non-normal data were log transformed prior to analysis. Two-factor ANOVA with Bonferroni post-hoc tests were used to describe genotype and exercise effects on protein levels, HDAC activity, and gene expression. Cardiac hypertrophy, tibia length, and wet heart weight data were resistant to transformations of normality and were analyzed with Kruskal-Wallis analysis of variance followed by Dunn’s post-hoc test. Values are presented as mean ± SEM and significance was accepted at P < 0.05.

## Results

### *Db* mice show obesity, hyperglycemia, and cardiac hypertrophy despite 4 weeks of exercise

**Figure 1:**
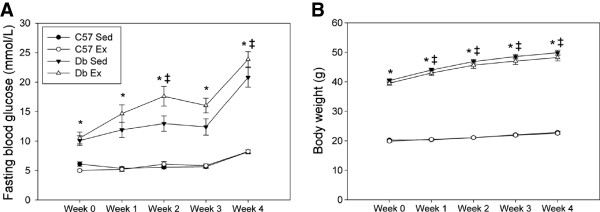
**Effect of 4 weeks of treadmill exercise training on fasting blood glucose and body weight in C57BL/6J (C57) and *db/db *(*db*) mice.** Ex = exercise (n = 15 per group); Sed = sedentary (n = 16 per group). * Significant effect of genotype within a time point, p < 0.05. ǂ Significant effect of time from previous consecutive time point, P < 0.05.

Fasting blood glucose and body weight in *db* mice were significantly elevated relative to controls, and increased over the duration of the protocol (Figure 1) as the *db* mice developed overt diabetes. Exercise did not alter either variable in *db* mice; this is consistent with previously described work by others [27,48,49]. There were no differences in cardiac hypertrophy at 1 week; however, *db* mice showed overt cardiac hypertrophy (heart weight:tibia length) at the 4 week time point (Table 3). Tibia length was also significantly reduced in *db* mice at the 4 week time point, consistent with previous reports of reduced linear skeletal growth in *db* mice [50,51].

**Table 3:** Morphological data

	**1 Week**			
	Wet heart (mg)	Tibia (mm)	Heart:tibia (mg:mm)	N
C57 Sed	115 ± 4	22.5 ± 0.2	5.13 ± 0.18	11
C57 Ex	111 ± 4	23.0 ± 0.2	4.83 ± 0.18	12
Db Sed	114 ± 3	22.4 ± 0.1	5.08 ± 0.14	11
Db Ex	116 ± 3	22.3 ± 0.2	5.20 ± 0.15	12
	*4 Week*			
	Wet heart (mg)	Tibia (mm)	Heart:tibia (mg:mm)	N
C57 Sed	113 ± 2	23.5 ± 0.1	4.82 ± 0.09	16
C57 Ex	105 ± 2	23.4 ± 0.1	4.50 ± 0.10	15
Db Sed	123 ± 4*	22.6 ± 0.1*	5.45 ± 0.15*	16
Db Ex	119 ± 3*	22.4 ± 0.1*	5.30 ± 0.13*	15

*p < 0.05, significant effect of genotype.

Results are presented as mean ± SEM.

### Total protein O-GlcNAcylation is increased by exercise in *db* mouse hearts

**Figure 2:**
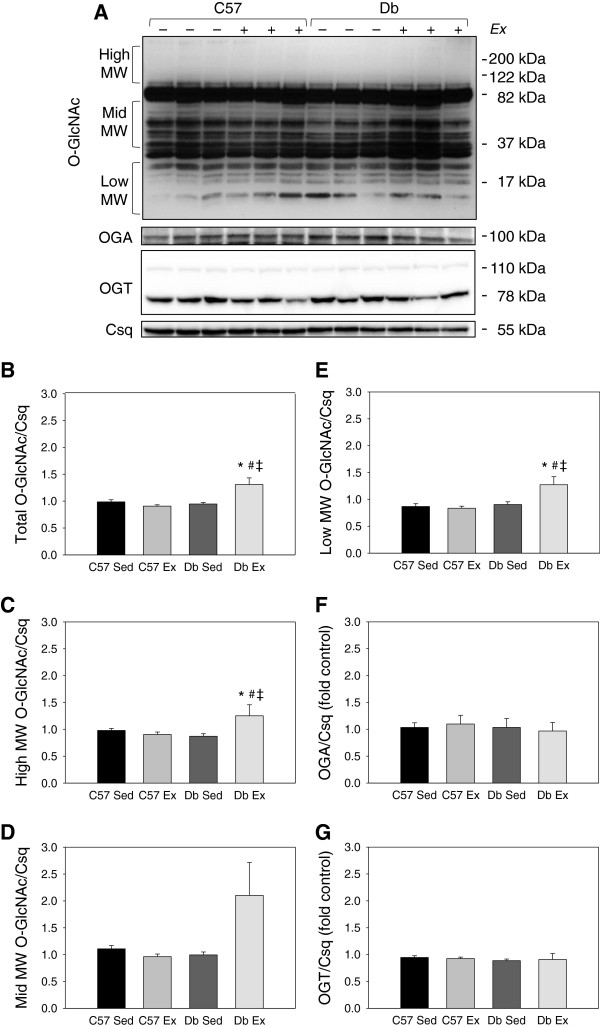
**1 week of exercise increases total protein O-GlcNAcylation in *db/db *mouse hearts. (A)** Western blot and **(B-G)** quantification of total protein O-GlcNAcylation in ventricular tissue from C57BL/6J (C57) and *db/db* (*db*) mice that were exercised (Ex) or sedentary (Sed) for 1 week. O-GlcNAc levels are normalized to the loading control calsequestrin (Csq). N = 6 per group. * Significant effect of genotype within an exercise group, P < 0.05. # Significant effect of exercise within a genotype group, P < 0.05. ‡ Significant interaction of exercise x genotype, P < 0.05.

**Figure 3:**
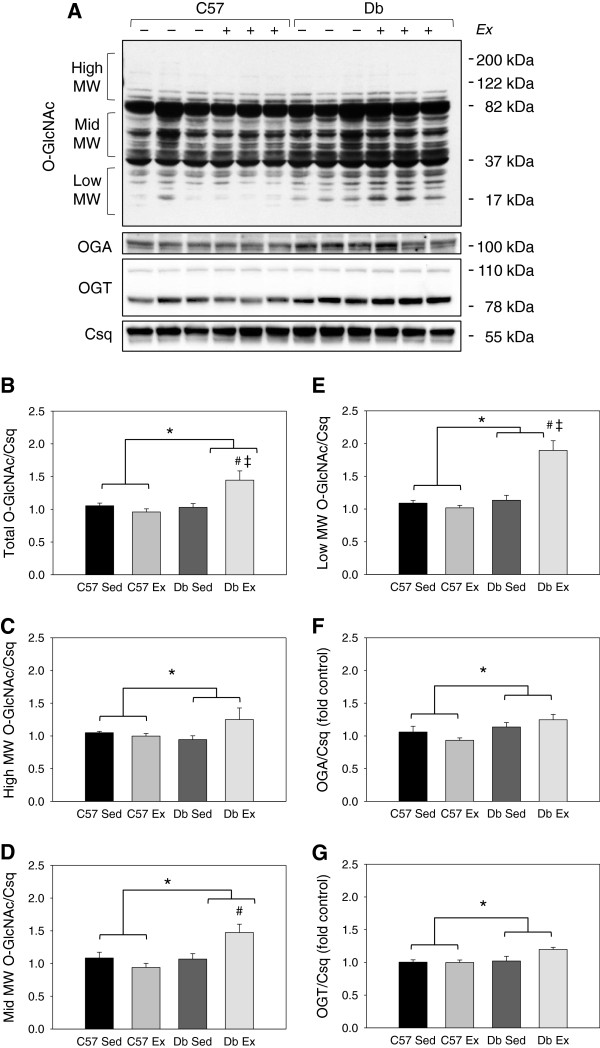
**4 weeks of exercise increases total protein O-GlcNAcylation in *db/db *mouse hearts. (A)** Western blot and **(B-G)** quantification of total protein O-GlcNAcylation in ventricular tissue from C57BL/6J (C57) and *db/db* (*db*) mice that were exercised (Ex) or sedentary (Sed) for 1 week. O-GlcNAc levels are normalized to the loading control calsequestrin (Csq). N = 6 per group. * Significant main effect of genotype, P < 0.05. # Significant effect of exercise within a genotype group, P < 0.05. ‡ Significant interaction of exercise x genotype, P < 0.05.

Exercise training increased cardiac O-GlcNAc in *db* mouse hearts at the 1 week time point; however, at this time point, protein levels of OGT and OGA were not different between groups (Figure 2). At 4 weeks, O-GlcNAc was elevated in *db* hearts relative to controls, and was significantly increased by exercise (Figure 3). As densitometry of the entire sample lane is dominated by the intense immunoreactive bands at 37 and 82 kDa, the analysis was also performed over the high, mid and low molecular weight ranges, which showed the same exercise-induced increases in *db* hearts. At this timepoint, levels of OGA and OGT were also significantly increased in *db* hearts relative to controls, independent of exercise.

**Figure 4:**
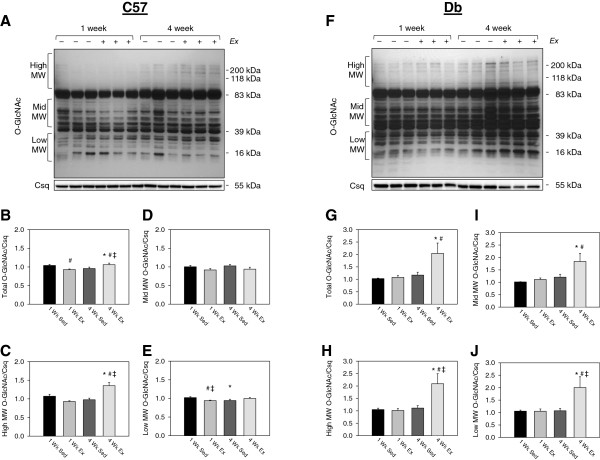
**Effects of 1 and 4 weeks of treadmill exercise on total protein O-GlcNAcylation in type 2 diabetic *db/db *and non-diabetic C57BL/6J hearts. (A)** Western blot and **(B-E)** quantification of total protein O-GlcNAcylation in ventricular tissue from C57 mice that were treadmill exercised (Ex) or sedentary (Sed) for 1 or 4 weeks. **(F)** Western blot and **(G-J)** quantification of protein O-GlcNAcylation levels in ventricular tissue from Ex or Sed (*db*) mice. O-GlcNAc levels are normalized to the loading control calsequestrin (Csq). N=6 per group. * Significant effect of time within an exercise group, P<0.05. # Significant effect of exercise within a time point, P<0.05. ‡ Significant interaction of exercise group x time, P<0.05.

To analyze these effects further, we then analyzed O-GlcNAc by weight range within each genotype. Analysis of O-GlcNAc by weight range revealed a modest but significant decrease in total protein O-GlcNAcylation with exercise. Interestingly, however, we also observed an increase in O-GlcNAc on total and high molecular weight proteins over time in C57 hearts (Figure 4C). In *db* mice, however, total and high molecular weight O-GlcNAc increased over time (Figure 4G), and O-GlcNAcylation of mid and low molecular weight were increased by exercise (Figure 4I-J).

### Diabetes reduces mSin3A/HDAC1/HDAC2 protein levels in the heart

**Figure 5:**
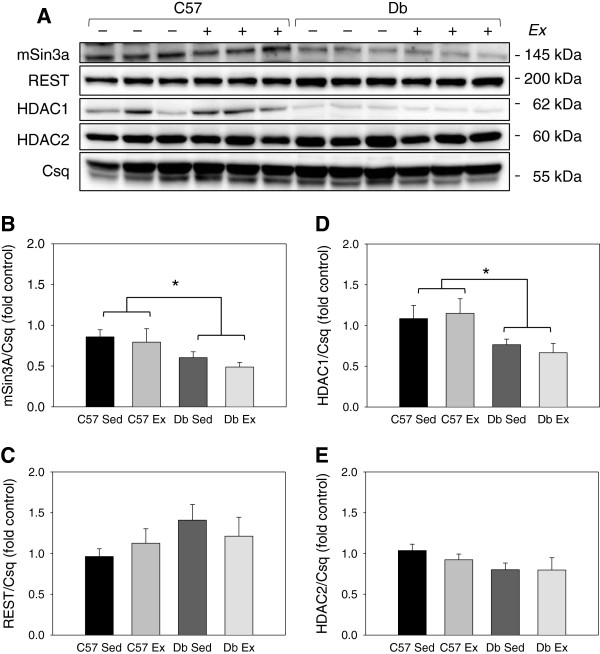
**Effects of diabetes and 1 week of exercise on protein levels of the mSin3A/HDAC1/2 transcription factor complex in type 2 diabetic *db/db *and non-diabetic C57BL/6J hearts. (A)** Western blot and **(B-E)** quantification of members of the mSin3A/REST/HDAC1/2 complex in C57BL/6J (C57) and *db/db* (*db*) mice who were treadmill exercise trained (Ex) or sedentary (Sed) for 1 weeks. N = 6 per group. * Significant main effect of genotype, P < 0.05.

**Figure 6:**
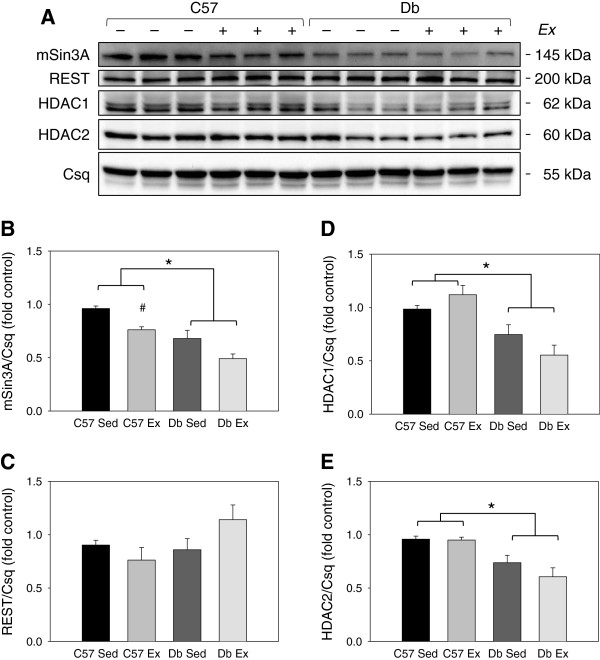
**Effects of diabetes and 4 weeks of exercise on protein levels of the mSin3A/HDAC1/2 transcription factor complex in type 2 diabetic *db/db *and non-diabetic C57BL/6J hearts. (A)** Western blot and **(B-E)** quantification of members of the mSin3A/REST/HDAC1/2 complex in C57BL/6J (C57) and *db/db* (*db*) mice who were treadmill exercise trained (Ex) or sedentary (Sed) for 4 weeks. N=6 per group. * Significant main effect of genotype, P<0.05; # Significant effect of exercise within a genotype group, P<0.05.

REST protein levels were not different between groups at either time point (Figures 5 and 6). At the 1 week time point, mSin3A and HDAC1 were significantly lower in *db* hearts, independent of exercise (Figure 5). At the 4 week time point, mSin3A, HDAC1, as well as HDAC2 protein levels were significantly lower in *db* hearts, again independent of exercise (Figure 6). Exercise training reduced mSin3A levels in non-diabetic control hearts; however, mSin3A levels were even lower in *db* hearts and did not change with exercise (Figure 6B).

### Exercise rescues the mSin3A:HDAC1 association and the OGT:HDAC2 association in *db* mouse hearts

**Figure 7:**
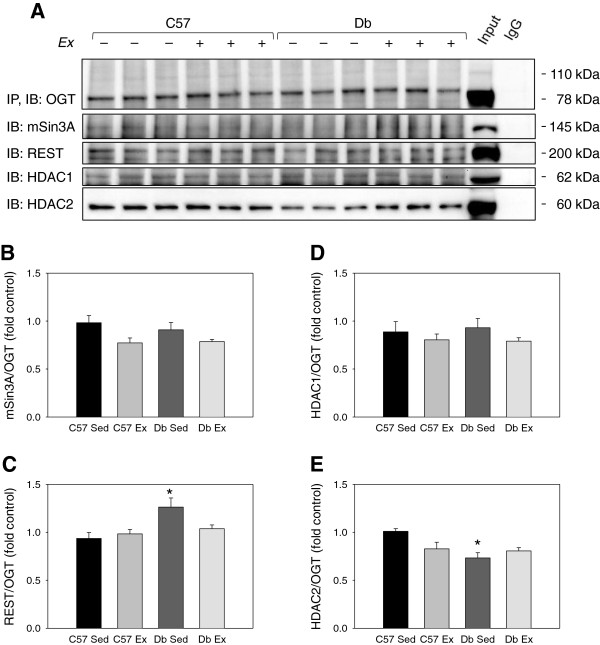
**Effects of diabetes and 4 weeks of exercise on the association of mSin3A/HDAC1/2 complex with OGT in type 2 diabetic *db/db *and non-diabetic C57BL/6J hearts. (A)** Immunoblot and **(B-E)** blot quantification showing the association of OGT with the mSin3A/REST/HDAC1/2 complex in C57BL/6J (C57) and *db/db* (*db*) mice who were treadmill exercise trained (Ex) or sedentary (Sed) for 4 weeks. N = 3 per group. * Significant effect of genotype within exercise group, P < 0.05.

As stated above, cardiac hypertrophy as measured by heart weight:tibia length was not apparent at 1 week but was evident in *db* hearts at 4 weeks (Table 3); therefore, we analyzed the association of hypertrophy-regulating transcription factors in the 4 week group only. Co-immunoprecipitation of OGT showed that diabetes reduced the OGT:HDAC2 association (P < 0.05), but this difference was removed by exercise training (Figure 7). OGT association with mSin3A and HDAC1 was not different between groups. OGT was also modestly more associated with REST in sedentary *db* hearts compared to sedentary controls (P < 0.05).

**Figure 8:**
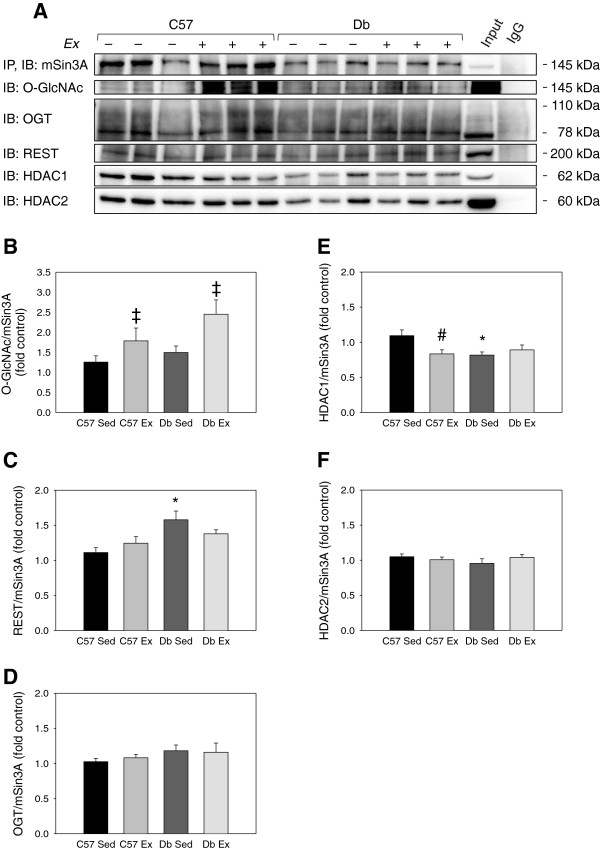
**Effects of diabetes and 4 weeks of exercise on the interaction of mSin3A/HDAC1/2 with OGT and O-GlcNAc in type 2 diabetic *db/db *and non-diabetic C57BL/6J hearts. (A)** Immunoblot and **(B-F)** blot quantification showing the O-GlcNAc modification and the association of the mSin3A/REST/HDAC1/2 complex with OGT in C57BL/6J (C57) and *db/db* (*db*) mice who were treadmill exercise trained (Ex) or sedentary (Sed) for 4 weeks. N = 6 per group. * Significant effect of genotype within exercise group, P < 0.05. # Significant effect of exercise within a genotype group, P < 0.05. ‡ Significant main effect of exercise, P < 0.05.

Reciprocal co-immunoprecipitation of mSin3A showed that there were no differences in its association with OGT or HDAC2 (Figure 8). However, mSin3A was significantly more associated with REST in *db* hearts. Finally, the association of mSin3A with HDAC1 was significantly lower in sedentary *db* hearts compared to sedentary controls, and this difference was removed by exercise.

It was recently reported that exercise reduces the O-GlcNAcylation of Specificity Protein 1 transcription factor (SP1) [43,44]. Therefore, we investigated whether exercise would alter the O-GlcNAc modification of mSin3A, a transcription factor that is directly involved in hypertrophic signaling. Although mSin3A immunoprecipitation appeared to show that the O-GlcNAcylation of mSin3A was increased by exercise (Figure 8A-B), reciprocal immunoprecipitation of O-GlcNAc did not confirm this effect (data not shown). Therefore, this effect of exercise should be viewed with caution.

**Figure 9:**
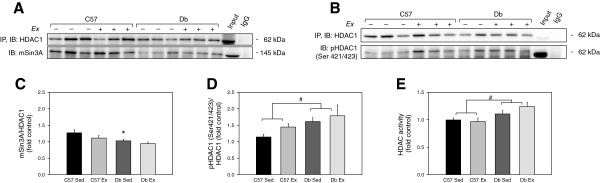
**Effects of diabetes and 4 weeks of exercise on HDAC1 activation and the mSin3A/HDAC1 interaction. (A)** Immunoprecipitation (IP) of HDAC1 and immunoblot for its association with mSin3A in ventricular tissue from C57BL/6J (C57) and *db/db* (*db*) mice that were treadmill exercised (Ex) or sedentary (Sed) for 4 weeks*.***(B)** Immunoprecipitation (IP) of HDAC1 and immunoblot for phospho-serine 421/423, which activates HDAC1. **(C-D)** Blot quantification of **(A)** and **(B)**. **(E)** Colorimetric assay quantification of class 1 HDAC activity in ventricular tissue. N = 6 per group for HDAC1 immunoprecipitation; N = 3 per group for HDAC1/2 activity assay. * Significant effect of genotype within an exercise group, P < 0.05. # Significant main effect of genotype, P < 0.05.

Finally, we performed co-immunoprecipitation of HDAC1 to confirm the results of the mSin3A immunoprecipitation (Figure 9A). Immunoblotting for mSin3A showed the same effect of exercise on the HDAC1:mSin3A interaction; the association of mSin3A with HDAC1 was lower in sedentary *db* hearts relative to sedentary C57 controls, but exercise removed this difference.

### HDAC activity is increased in *db* mouse hearts

Phosphorylation of HDAC1 at serine 421 and 423 is specifically associated with HDAC1 activation [52]; therefore, we immunoprecipitated HDAC1 and immunoblotted for phospho-HDAC1 (Ser421/423). This showed that HDAC1 phosphorylation was significantly elevated in *db* hearts independent of exercise (P < 0.05) (Figure 9D). Colorimetric assay confirmed that class I HDAC activity was significantly higher in *db* mice independent of exercise (Figure 9E).

### Diabetes blunts fetal gene expression in the heart

**Figure 10:**
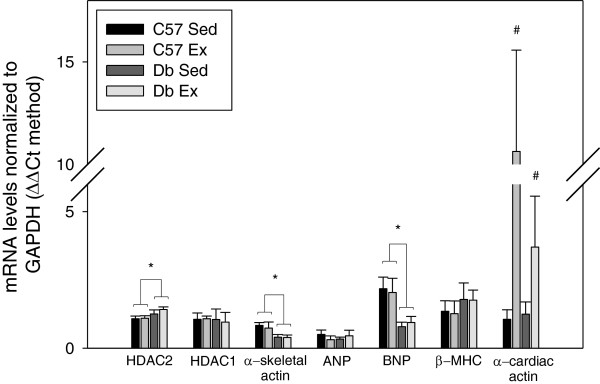
**mRNA transcript levels in ventricular tissue from C57BL/6J (C57) or *db/db *(*db*) mice that were treadmill exercised (Ex) or sedentary (Sed) for 4 weeks.** mRNA levels are normalized to the loading control GAPDH using the 2^-∆∆Ct^ method. N = 8 per group for cardiac α-actin; N = 6 all other groups. * Significant main effect of genotype, P < 0.05. # Near-significant main effect of exercise, P_trend_ = 0.050.

We investigated whether changes in these transcription factor interactions were associated with changes in the expression of fetal genes, such as ANP, BNP, and skeletal α-actin, which are regulated by REST and mSin3A [20,53]. Transcript levels of ANP and β-myosin heavy chain were not different between groups, but BNP and skeletal α-actin were significantly reduced in *db* hearts independent of exercise (Figure 10). HDAC2 gene expression was elevated in *db* hearts independent of exercise, and cardiac α-actin showed a prominent trend to be induced by exercise (P = 0.050) (Figure 10).

## Discussion

While diabetes is a multifactorial cardiac insult, and diabetic cardiomyopathy is associated with multiple factors such as oxidative stress [54], lipotoxicity and mitochondrial dysfunction [55-57], and impaired calcium signaling [13], O-GlcNAc is emerging as an important signalling mechanism in the development of diabetic cardiomyopathy. Total protein O-GlcNAcylation is chronically elevated in the type 1 and 2 diabetic heart [4,39], and reducing protein O-GlcNAcylation by adenoviral overexpression of OGA [38] improves cardiac function. Similarly, lowering O-GlcNAc by intensive swim training [43,44] has been proposed as a mechanism by which exercise benefits the diabetic heart, and exercise lowers both the O-GlcNAc modification of the SP1 transcription factor and the OGT enzyme. O-GlcNAc directly mediates the expression of fetal genes in response to hypertrophic stimuli [29], and O-GlcNAc modifies mSin3A and HDAC1 [35], which regulate cardiac hypertrophy [20,58]. Previously, we have shown that exercise lowers the O-GlcNAc modification of the OGT enzyme [32], and others have shown that exercise lowers O-GlcNAcylation of the SP1 transcription factor [43]. Moderate exercise improves cardiac structure and function in humans with type 2 diabetes [59,60]; we therefore tested the hypothesis that moderate exercise would reduce O-GlcNAc in the type 2 diabetic heart, and would be associated with changes in the O-GlcNAc modification and activity of the mSin3A/HDAC1/2 transcription factor complex, which regulates hypertrophic genes.

Surprisingly, and in contrast with the previous studies, we found that 4 weeks of moderate treadmill exercise increased total O-GlcNAc in type 2 diabetic *db* mouse hearts. Also, while the previous studies showed that OGT was also reduced by exercise [43,44], we found that OGT and OGA expression was elevated in *db* hearts and did not change with exercise. Such parallel regulation of OGT and OGA expression has been previously reported [41], and may represent a compensatory relationship between these two opposing enzymes. The difference in our findings may be due to the use of type 2 *db* mice rather than streptozotocin-induced type 1 diabetic mice, and the use of moderate treadmill exercise rather than more intensive swimming exercise. However, other studies have shown that an upregulation of O-GlcNAc is essential in the cardiac stress response [61,62], is acutely cardioprotective [63,64], and is part of a constitutively active cardioprotection mechanism in the diabetic myocardium [42]. Therefore, these data suggest that an increase in cardiac O-GlcNAc in the type 2 diabetic heart may be a beneficial effect of exercise.

In our study, mSin3A immunoprecipitation revealed that exercise increased the O-GlcNAc modification of mSin3A; however, this was not supported by reciprocal O-GlcNAc immunoprecipitation. It is possible that the large amount of protein captured in the O-GlcNAc immunoprecipitation masked the changes in mSin3A O-GlcNAcylation, which we observed in the more specific mSin3A immunoprecipitation. However, these data underscore the importance of verifying changes in O-GlcNAcylation of individual proteins with reciprocal assays, and suggest that moderate changes in protein O-GlcNAcylation – including those in the present study – should be interpreted cautiously and confirmed by additional studies.

Nevertheless, our data do suggest an alternate mechanism for the beneficial effect of exercise on the diabetic heart. *Db* hearts showed lower protein levels of mSin3A, HDAC1, and HDAC2, and an increased association of mSin3A with REST, independent of exercise. Likewise, mRNA transcript levels of BNP and α-skeletal actin, which are typical markers of cardiac hypertrophy activated by HDAC1/2 [65] that are regulated via REST/mSin3A [33], were significantly lower in *db* hearts independent of exercise. The finding that blunted expression of fetal genes in diabetic hearts is not altered by exercise has been shown in previous studies [27,28]. Therefore, we suggest that the loss of HDAC1/2 and the increased association of the mSin3A corepressor with REST may underlie the blunted expression of fetal genes in the diabetic heart. Further, since the natriuretic peptides are both anti-hypertrophic and cardioprotective [15,17,28], we suggest that this mechanism may be responsible for the increased vulnerability of the diabetic heart to stress and heart failure [66,67].

Although we did not measure the structural or hemodynamic effects of the exercise protocol in *db* hearts, previous work has shown that the *db* heart shows similar cardiomyopathy to humans with type 2 diabetes [68,69], which are improved by exercise [13,14]. We show additionally that even the low intensity of exercise used in this protocol was sufficient to elevate the expression of cardiac α-actin in C57 hearts (P = 0.050). Cardiac α-actin is a marker of cardiomyocyte differentiation and hypertrophy [70], and is increased in physiologically hypertrophied hearts after chronic endurance exercise training [25]. Additional transcriptional changes were observed in *db* hearts, in which the exercise protocol significantly increased the association of mSin3A and OGT with HDAC1 and HDAC2, respectively. Therefore, although the exercise stimulus used in this study did not cause overt changes in cardiac mass, it induced transcriptional events consistent with the early stages of physiological cardiac remodelling.

Finally, these data show a potential interaction between HDAC1 and HDAC2 that has not previously been described in the heart. HDAC1 and HDAC2 regulate cardiac hypertrophy in a similar manner [58], and HDAC1 deficiency induces HDAC2 expression in embryonic stem cells [71]. In our study, the loss of HDAC1 protein preceded the loss of HDAC2 protein in *db* hearts, and was similarly associated with an increase in HDAC2 gene expression in *db* hearts. When HDAC2 deficiency was present at the 4 week time point, we observed an increase in the total activity of class I HDACs in *db* hearts, which was verified by an increase in the phosphorylation status of HDAC1 at Ser421/423. Phosphorylation at these residues is specifically associated with HDAC1 activity [52]. Therefore, these data suggest that the class I HDACs have compensatory effects on each other’s expression levels and activation by phosphorylation. Further, the reduction in HDAC2 protein levels in *db* mouse hearts did not occur until 4 weeks, and was associated with overt cardiac hypertrophy. Thus, the loss of HDAC2 in the diabetic heart is associated with the progression of hypertrophy in the diabetic heart, and may be more specifically involved in hypertrophy than HDAC1.

## Conclusions

These data show that exercise increases O-GlcNAc in the type 2 diabetic *db* mouse heart, and that components of the mSin3A/HDAC1/2 chromatin-modifying complex interact with O-GlcNAc and OGT. Contrary to our hypothesis, exercise increased cardiac O-GlcNAc in the diabetic heart; this signalling mechanism may underlie the beneficial effect of exercise in the pathologically hypertrophied diabetic heart. Finally, we found that diabetes and exercise reciprocally affected the physical associations of mSin3A/HDAC1/2. The effects of exercise observed in this study were generally modest, which suggests that a moderate level of exercise, such as that prescribed for human patients with diabetes, does not have extreme effects on the mSin3A/HDAC1/2 complex. However, since this complex is a key regulator of cardiac hypertrophy, the results of this study suggest that exercise-induced changes in the association or activity of this complex may underlie the beneficial effect of moderate exercise in the diabetic heart.

## Abbreviations

ANP: Atrial natriuretic peptide; BNP: Brain natriuretic peptide; C57: C57 background strain control mouse; Db: db/db type 2 diabetic mouse; HDAC: Histone deacetylase; mSin3A: Mammalian switch-independent 3A; O-GlcNAc: O-linked β-N-acetylglucosamine; OGT: O-GlcNAc transferase; OGA: O-GlcNAcase; REST: Repressor element-1 silencing transcription factor.

## Competing interests

The authors declare that they have no competing interests.

## Authors’ contributions

EJC performed the animal studies and laboratory experiments, and drafted the manuscript. SAM edited the manuscript and provided technical and intellectual guidance. Both authors read and approved the final manuscript.
